# Research on Odor Interaction between Aldehyde Compounds via a Partial Differential Equation (PDE) Model

**DOI:** 10.3390/s150202888

**Published:** 2015-01-28

**Authors:** LuchunYan, Jiemin Liu, Chen Qu, Xingye Gu, Xia Zhao

**Affiliations:** School of Chemistry and Biological Engineering, University of Science and Technology Beijing, Xueyuan Road 30, Haidian District, Beijing 100083, China; E-Mails: yanluchun@126.com (L.Y.); quchen5626@163.com (C.Q.); gxy93@126.com (X.G.); zhaoxia0719@163.com (X.Z.)

**Keywords:** human sensing, sensory evaluation, odor intensity, odor interaction, air pollution

## Abstract

In order to explore the odor interaction of binary odor mixtures, a series of odor intensity evaluation tests were performed using both individual components and binary mixtures of aldehydes. Based on the linear relation between the logarithm of odor activity value and odor intensity of individual substances, the relationship between concentrations of individual constituents and their joint odor intensity was investigated by employing a partial differential equation (PDE) model. The obtained results showed that the binary odor interaction was mainly influenced by the mixing ratio of two constituents, but not the concentration level of an odor sample. Besides, an extended PDE model was also proposed on the basis of the above experiments. Through a series of odor intensity matching tests for several different binary odor mixtures, the extended PDE model was proved effective at odor intensity prediction. Furthermore, odorants of the same chemical group and similar odor type exhibited similar characteristics in the binary odor interaction. The overall results suggested that the PDE model is a more interpretable way of demonstrating the odor interactions of binary odor mixtures.

## 1. Introduction

Odor pollution usually causes serious annoyance in urban areas because of the pungent smell and health hazards [1]. Usually, the composition of odor pollutants and their concentrations are various in different fields. Because the odor interactions among these pollutants distinctly influence the odor qualities (e.g., odor intensity, odor type and hedonic tone) of their mixtures, sensory evaluation is always employed to make a more intuitive assessment of the odor pollution instead of instrumental analysis [2,3]. The odor intensity (OI) in particular is used as an index for the evaluation of air pollution [4]. However, the application of sensory evaluation is normally limited by the needs for experienced assessors and an odor-free testing environment. Thus, the relationship between the OI of a perception and the concentrations of stimuli needs to be carefully explored [5].

The odor interactions of odorants have been divided into several kinds of effects, including synergism, antagonism, averaging, *etc.* [6,7]. Based on that, the OI of a mixture is modeled by assuming an aggregation of individual constituents' contributions. At present, several models are proposed and they focus on linking the perceived OI of a mixture to the individual odor intensities of its unmixed components [8]. For example, the Strongest Component Model calculated the OI of a mixture as that of the strongest of the unmixed components; the Vector Model calculated the OI of a mixture as the vector sum of the OI of its unmixed components; the U Model was proposed as a modified version of Vector Model to overcome its inability to predict hyperadditivity [9]. However, the OI of these unmixed components in the above mentioned models are still rated by sensory panels. In a previous study, the odor interaction of a binary odor mixture was investigated by referencing the partial molar volume measurement method [10]. After displacing the key parameters of partial molar volume calculating formulas with suitable perceptual measures, a partial differential equation (PDE) model was proposed. Through a series of odor intensity matching tests for binary mixtures of benzene and substituted benzenes, the PDE model was proved effective at OI prediction. Besides, it also provided a more interpretable way of relating the concentration of each component to their joint OI. Thus, OI of a binary odor mixture can be predicted without the participation of human assessor.

In this paper, several aldehyde compounds with similar molecular structure were chosen as the target substances. Three binary mixtures of aldehydes were prepared in the form of a series of different odor samples, and a PDE model was respectively established for each odor mixture. The effects of concentration level and random error on the PDE model were carefully investigated. Based on these experiments, an extended PDE model was established. Through a series of odor intensity matching tests, we discussed the similarity of odorants with same chemical group and similar odor type in the performance of binary odor interaction.

## 2. Materials and Methods

### 2.1. Samples and Sensory Evaluation Methods

Odor samples of both individual aldehydes (Table 1) and their binary mixtures were prepared using chemicals purchased from J&K Scientific (Beijing, China). Each odor sample was prepared through transferring a certain amount of standard gas to an odor-free plastic bag (Sinodour, Tianjin, China) and diluted with purified air [10].

Fourteen assessors (mean age: 26 years, range: 20–37) were recruited from the University of Science and Technology Beijing. All of them had previous experience evaluating the odor intensity of odor samples. The assessors were grouped into panel A (four males and four females) and panel B (three males and three females). Panel A participated in the tests of PDE model establishment, and panel B was employed in the odor intensity matching tests. Before tests, odor intensity references were firstly prepared according to the ASTM odor intensity referencing scale (OIRS, water solutions of 1-butanol from level 1(aqueous solution of 12 ppm) to level 8 (1550 ppm) with a geometric progression of two at 27 ± 1 °C [13]. During the sensory evaluation, each assessor gently pushed the odor sample bag and simultaneously sniffed at the sniffing pipe (inner diameter of 1 cm) on the bag. Then, assessors sniffed the previously prepared odor intensity references and they were asked to point the best match on the OIRS which exhibited an odor intensity matching that of the sample. If the best match on the scale was a position between two continuous scale points, then a half number (e.g., 4.5) was also accepted. Each odor sample was evaluated by a sensory panel and the mean value of assessors' rating results was calculated as the measured odor intensity (*i.e.*, Measured OI).

Odor threshold was a fundamental parameter in this study, and the odor threshold of each odorant was measured by assessors of panel A. Odor samples of an odorant were delivered by a dynamic olfactometer (AC'SCENT, St. Croix Sensory, Inc., Stillwater, MN, USA) in an ascending dilution series, and assessors sniffed from the sniffing mask with one presentation of dilute odor and two blank presentations in turn. Between two continuous dilution levels, about 30 s of interval time was arranged to avoid the olfactory fatigue. The final correctly identified dilution level of each assessor was recorded, and the odor threshold was calculated from the initial concentration of the odorant and average of the sensory panel's recorded dilution multiples. As shown in Table 1, the measured odor thresholds and two sets of reported odor thresholds were listed. It was proposed that much of the variation of odor thresholds among laboratories is attributable to issues of experimental precision, especially concerning control of odorant concentration [14]. Because the measuring methods of each study were different (e.g., dynamic olfactometer method and triangle odor bag method), extremely large variation in odor thresholds was reported by different laboratories [11,12,15]. As listed in Table 1, our measured odor thresholds was within a normal range. Except for the odorants with side chain (*i.e.*, IBA and IVA in Table 1), our measured odor thresholds also basically followed the common law that the values of odor thresholds decrease along with the increase of carbon chain length. In this study, our measured odor thresholds were employed throughout the experiments. Because exploring the relation between two perceptual measures, it was considered more comparable when they were measured by the same group of assessors. Actually, the comparability of experimental results was more focused and researchers used to measure odor thresholds by themselves in their studies. For example, Karnekull *et al*. employed a group of participants to assess odor threshold, odor intensity, perceived odor hedonics and performed their regression models [16].

### 2.2. The PDE Model Methodology

**Figure 1.**
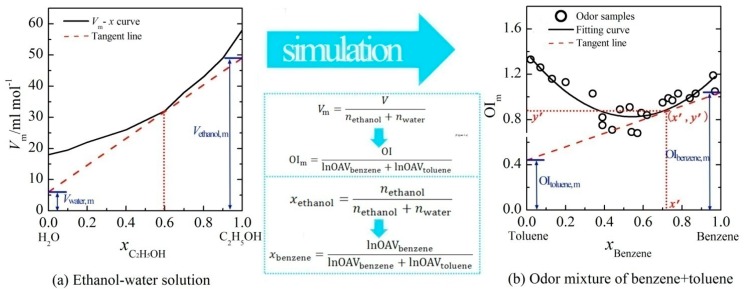
The (**a**) tangent-intercept method of partial molar volume; and (**b**) the methodology of PDE model for binary odor mixture by reference to it [10].

In a previous study, the linear relations between OI and logarithm of odor activity value (lnOAV) of individual aromatic odorants had been demonstrated [10]. Based on that, the key parameters of tangent-intercept method (*i.e.*, a common measurement method of partial molar volume) were suitably displaced with olfactory measures (Figure 1). Then, the PDE model was proposed in the form of a set of partial differential equations by reference to the partial molar volume measurement. The detailed equations were described as below:

(1) $${\text{OI}}_{\text{m}}=\text{OI}/(\ln{\text{OAV}}_{\text{a}}+\ln{\text{OAV}}_{\text{b}})$$

(2) $$x_{\text{a}}=\ln{\text{OAV}}_{\text{a}}/(\ln{\text{OAV}}_{\text{a}}+\ln{\text{OAV}}_{\text{b}})$$

(3) $${\text{OI}}_{\text{a},\text{m}}=f_{2}\left(1\right)=\left(1-x^{\prime }\right)\cdot f^{\prime }\left(x^{\prime }\right)+y^{\prime }$$

(4) $${\text{OI}}_{\text{b},\text{m}}=f_{2}\left(0\right)=-x^{\prime }\cdot f^{\prime }\left(x^{\prime }\right)+y^{\prime }$$

where *a* and *b* represent two different odorants; OI is the odor intensity of a binary odor mixture; OI_m_ is the averaged OI of each lnOAV unit odorant; *x*_a_ is the mixing proportion of substance *a*; OI_a,m_ (or OI_b,m_) is the partial differential OI of substance *a* (or *b*), which represents the OI of each lnOAV unit substance *a* (or substance *b*) actually contributed to the OI of whole binary odor mixture.

As shown in Figure 1, the volume of solution (*V*) was displaced by the OI of a binary mixture and the mole number (*n*) of a substance was displaced by the lnOAV of an odorant. Let OI_m_ be the vertical axis and *x* be horizontal axis (*i.e.*, Equations (1) and (2)), the experimental results of a binary mixture (e.g., benzene + toluene) were calculated and depicted in Figure 1b. Nonlinear fitting of these data was performed, and the corresponding curve-fitting equation was *y* = *f*(*x*). For an odor sample with a fixed mixing ratio (e.g., (*x′, y′*)), a tangent line (*i.e., y* = *f*_2_(*x*) in Equations (3) and (4)) was plotted at the corresponding point on the curve. The intercept of this tangent line on the vertical axis *x* = 1 (or *x* = 0) just was the value of OI_a,m_ (or OI_b,m_). Through observing the OI_a,m_ and OI_b,m_ in the PDE model, the effects of constituents' mixing ratio on the binary odor interaction could be deeply explored.

Based on the summation formula of partial molar volume:

(5) $$V=V_{1,\text{m}}\cdot n_{1}+V_{2,\text{m}}\cdot n_{2}$$

the OI of a binary odor mixture also could be calculated as the following formula:

(6) $$\text{OI}={\text{OI}}_{\text{a},\text{m}}\cdot \ln{\text{OAV}}_{\text{a}}+{\text{OI}}_{\text{b},\text{m}}\cdot \ln{\text{OAV}}_{\text{b}}$$

Through the quantitative analysis of a binary odor mixture, its OI could be directly predicted by employing the PDE model.

### 2.3. Experimental Scheme

**Figure 2.**
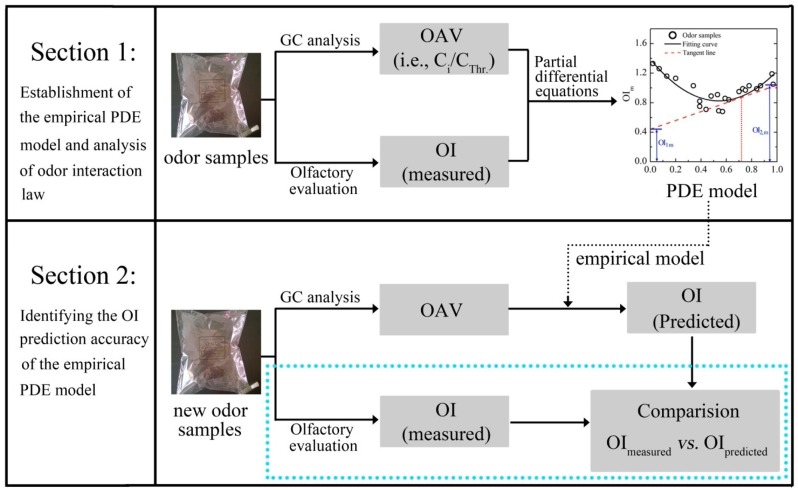
Experimental scheme of the two sections for (**1**) the establishment of PDE model and (**2**) the confirmation of its odor intensity predictive performance.

The experimental scheme of this study is basically presented in Figure 2. It was mainly divided into two major sections. In the first section, the odor interaction of binary odor mixtures was explored by employing a set of partial differential equations and the specific PDE model was individually established for each binary mixture. At first, each odorant (*i.e.*, PA, VA and HEP) was prepared as five odor samples with odor intensities evenly ranging from level 1 to level 6 of the OIRS. Each odor sample was rated by panel A and its concentration was identified by GC-FID analysis. Then, the linear relation between OI and concentration of individual odorant was demonstrated. After that, three binary mixtures of aldehydes (*i.e.*, mixture of PA and VA; PA and HEP; VA and HEP) were individually tested. Each binary mixture was consisted of 24 odor samples with various mixing ratios and different concentration levels. In order to avoid too strong olfactory stimulation, all the odor samples were prepared below 7.0 of the OIRS. According to that, the range of each odorant's chemical concentration in this test was determined as following: PA, 0.05–1.80 mg/m^3^; VA, 0.02–1.10 mg/m^3^; HEP, 0.03–1.20 mg/m^3^. The OI of each odor sample was rated by panel A, and the concentrations of its constituents were identified by GC-FID. After that, these results were calculated according to the above partial differential equations (Equations (1)–(4)). Then, a PDE model was individually established for each odor mixture. Through the analysis of each PDE model, the characteristics of odor interaction between two aldehyde compounds were suitably discussed. Especially, the effects of odor sample's concentration level and random error on odor interaction were explored in more detail.

In the second section, experimental data of the above three binary mixtures were gathered and these data were calculated to establish an extended PDE model. After that, a new set of binary odor samples were prepared. The OI of each odor sample was firstly rated by panel B. After measuring the concentrations of each sample's constituents, the partial differential OI of each substance was calculated following the extended PDE model. Then, the predicted odor intensity of each odor sample was also obtained according to Equation (6). Finally, the predictive performance of the PDE model was confirmed by comparing the measured OI of each odor sample with its corresponding predicted OI.

For an odor sample with known chemical concentration (C_i_) and odor threshold (C_Thr._), its OAV was calculated as: OAV = C_i_/C_Thr._. The numerical value of OAV was close to the dilution factor that was necessary to reach the odor threshold, and it indirectly represented the concentration level of an odor sample. The concentrations of odor samples in the above experiment were measured by a gas chromatography (GC-2014, Shimadzu, Kyoto, Japan) with a flame ionization detector (GC-FID). The Rtx-5 capillary column (30 m × 0.25 mm ID, 0.5 μm film thickness) was employed and the carrier gas was nitrogen (≥99.999%) at 1.0 mL/min. The injection port of gas chromatography was 200 °C. The column oven temperature was set to 50 °C for 3 min and up to 200 °C at 10 °C·min^−1^and held for 5 min.

## 3. Results and Discussion

### 3.1. The Linear OI-lnOAV Relation of Individual Aldehyde

**Figure 3.**
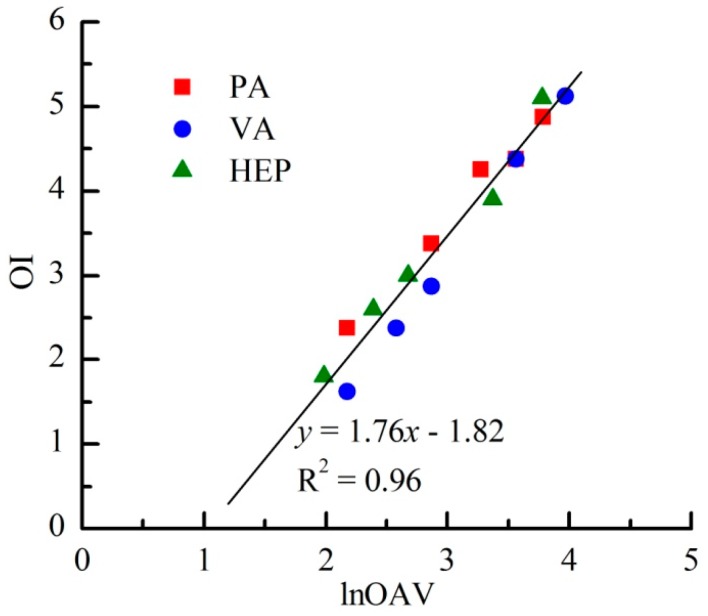
Odor intensity as a function of the concentration of the individual odorant.

As shown in Figure 3, OI linearly increased with the increasing lnOAV of individual odorant. As reported by Kim [17], a distinct linear relation between odor intensity and log (D/T) (*i.e.*, logarithm of dilution-to-threshold (D/T) ratio) also had been observed among four individual reduced sulfur compounds. The D/T ratio meant the dilution multiple of an odor sample when it was diluted to odorless. Because OAV was usually calculated as the ratio between the concentration of an individual substance and its threshold concentration (*i.e.*, the minimal concentration that can be detected by human nose), the value of D/T ratio and OAV was theoretically the same for an individual odor sample. Based on the above results, the linear relation between OI and lnOAV generally existed among individual odorants.

Besides the apparent linear OI-lnOAV relation of each individual odorant, all odor samples of PA, VA and HEP followed a same fitting equation (Figure 3). It probably indicated that each lnOAV unit of any odorant among these tested aldehydes always generated the same intensity of olfactory stimulation. With the same chemical group and similar molecular structure, the odor types of PA, VA and HEP were also almost the same (*i.e.*, “crushed grass” note) [18]. Usually, odorants with same functional group and similar molecular structure would have a similar odor type. For instance, acetic acid, butyric acid, hexanoic acid and octanoic acid all had a rancid fat note. However, maple lactone (“maple syrup” aroma) was quite different in odor type from the above mentioned carboxylic acids only because of its different molecular structure [19]. On the level of theory, Mori *et al*. have also systematically explained the relationship between odorant molecular features and perceived ‘odor’ from two aspects: the polar functional groups and the molecular profile [20]. Thus, the similarity of OI-lnOAV relations among aldehydes was possibly related with their odor type. With the same OI-lnOAV fitting equation, the laws of odor interactions among binary mixtures of PA, VA and HEP also tended to the same. Previous study has reported that binary mixtures of benzene, toluene, ethyl benzene and *o*-xylene could fit into a same extended PDE model on the basis of their similar OI-lnOAV relations [10].

### 3.2. The Odor Interaction of Binary Odor Mixture

#### 3.2.1. The PDE Model of Specific Odor Mixture

**Figure 4.**
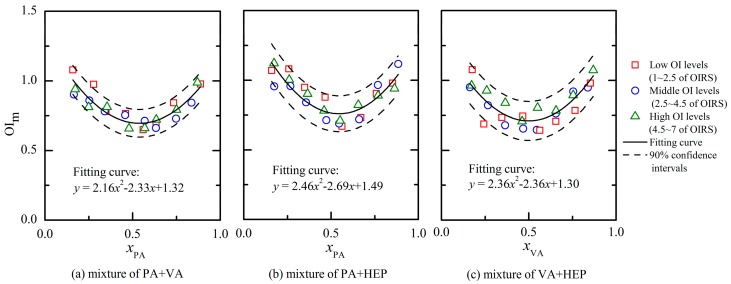
PDE models for binary mixture of: (**a**) propionaldehyde and *n*-valeraldehyde (PA + VA); (**b**) propionaldehyde and *n*-hexaldehyde (PA + HEP); (**c**) *n*-valeraldehyde and *n*-hexaldehyde (VA + HEP). The fitting curve of quadratic polynomial regression analysis (solid line) and corresponding confidence intervals (dashed line, confidence coefficient was 0.90) are plotted.

Figure 4 depicts the PDE models for three different binary odor mixtures of aldehydes. Every binary mixture had 24 different odor samples and the OI of each odor sample was rated by assessors of panel A. The PDE model was established according to the measured results (e.g., OI, chemical concentration and measured odor threshold in Table 1) and corresponding partial differential equations (*i.e.*, Equations (1)–(4)). The overlap of different odor samples' results existed in Figure 4, and it probably influenced the observed quantity of each mixture's odor samples. Besides all the odor samples were prepared with OI below 7.0 of the OIRS (*i.e.*, avoiding too strong olfactory stimulation), the mixing ratio of odorants (*i.e.*, horizontal axis in Figure 4) was also set between 0.1 and 0.9. In a previous study, it has confirmed that the presence of an odorant barely influenced the OI of a mixture when its lnOAV value was lower than 20% of the mixture's total amount [21]. Thus, odor samples with mixing ratio beyond the range of 0.2–0.8 were almost not investigated in this study. After the quadratic polynomial regression analysis of each binary odor mixture's results, the fitting curve showed an asymmetric “U” shape (Figure 4). Because the OI was rated by an olfactory method, the fluctuation of test results generally existed. Thus, odor samples dispersed within the confidence intervals of the fitting curve and it was considered to be random errors [10]. However, differences of fitting effect were still observed among odor mixtures that were comprised of components with various hydrophobic properties. For example, the binary mixture (Figure 4a, a mixture of PA + VA) that was comprised of components with low hydrophobicity produced the best fits; the binary mixture (Figure 4c, a mixture of VA + HEP) that was not as well fitted by modeling contained a component with high hydrophobicity (*i.e.*, HEP). According to the reported references, odorant deposition pattern in the nasal cavity played an important role in olfaction and it was seriously influenced by the odorant solubility in the mucus layer [22,23]. Low hydrophobicity was helpful to accelerate the adsorption process in the mucus layer and then improve the receptor sensitivity to the odorant. Thus, the above mentioned fluctuation of test results was more apparent when a mixture was comprised of components with high hydrophobicity (Figures 4b,c).

The researches of partial molar volume had proved that the value of a substance's partial molar volume was only determined by its mixing ratio in the solution. However, the partial molar volume was not influenced by the solution volume at all. Because the PDE model was established by referencing to the partial molar volume methodology, the influence of an odor mixture's OI on the OI_i,m_ (i stands for an odorant) values was also explored. At first, the 24 odor samples of each binary mixture were equally divided into three groups (eight odor samples of each group) on the basis of their corresponding measured OI (e.g., measured OI within 1–2.5 of OIRS was defined as low OI level; 2.5–4.5 of OIRS was middle OI level; 4.5–7 was high OI level). As shown in Figure 4, odor samples of three different OI levels were observed in a random distribution within the confidence intervals. Besides, the odor samples of a specific binary mixture were all in accordance with the same fitting curve no matter which the OI level it was. Thus, the measured OI_i,m_ values of a binary odor sample with a fixed mixing ratio were always constants at any concentration levels. Based on the above results, odor interaction of a binary mixture was mainly influenced by its mixing ratio instead of its concentration level.

#### 3.2.2. The Precision of OI Evaluation

**Figure 5.**
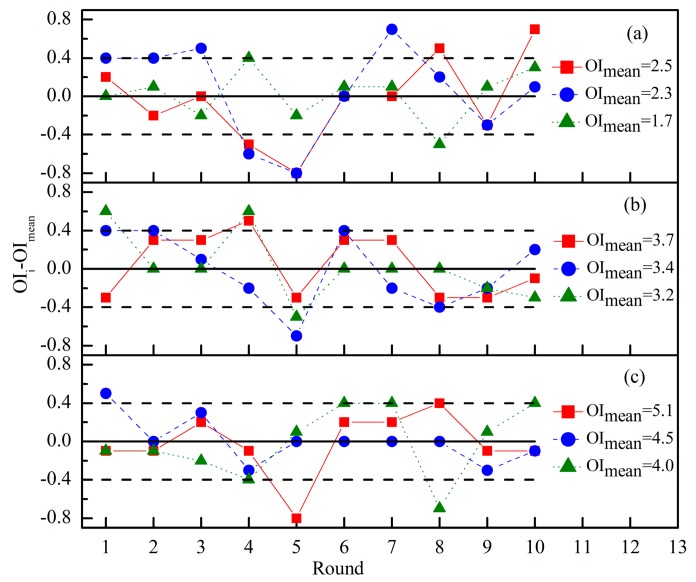
The precision of odor intensity evaluation in repeated tests for nine different odor samples of propionaldehyde (PA) and *n*-valeraldehyde (VA) mixture at (**a**) low odor intensity level; (**b**) middle odor intensity level and (**c**) high odor intensity level.

Odor intensity is the perceived strength of an odor sensation by human nose. Because the olfactory evaluation was easily influenced by many factors including age, gender, environmental conditions and so on, the fluctuation of measurement results were widely recognized. In order to further confirm the OI evaluation precision of panel A in this study, nine odor samples of binary mixture PA + VA were prepared and the OI of each odor sample was repeatedly measured 10 times by the panel A. For each odor sample, the mean value of ten continuous rated OI was calculated (*i.e.*, OI_mea._). The difference between a specific OI and OI_mea._ was also calculated as the vertical axis (Figure 5). Odor samples with low OI level (Figure 5a) showed an apparent fluctuation in the repeated test, but the fluctuation decreased following the increase of odor sample's OI level. For example, the measured results of most odor samples were located within ± 0.4 on the vertical axis. It was seven times out of this range at low OI levels (Figure 5a), five times at middle OI levels (Figure 5b) and only three times at high OI levels (Figure 5c). Odor sample at low OI level had weak stimulation on human nose, and assessors were in trouble of locating it at an accurate location on the OIRS. However, both odor type and odor intensity of odor sample became more distinct at high OI level. It was helpful for assessors to successfully locate an odor sample on the OIRS. For an odor sample with OI_mea._ = 1.7 (Figure 5a), the total amount of two components' lnOAV was 2.2. Then, the normal fluctuation (0.4 of OIRS in Figure 5) would cause a difference of 0.18 between two corresponding OI_m_ values (Equation (1)). Following the increase of odor sample's OI level, the normal fluctuation caused difference between two OI_m_ values would even smaller. Based on the above results, the fluctuations of odor samples in Figure 4 really correspond to random errors.

### 3.3. The Similarities of Binary Odor Interaction of Aldehydes

#### 3.3.1. The Extended PDE Model

**Figure 6.**
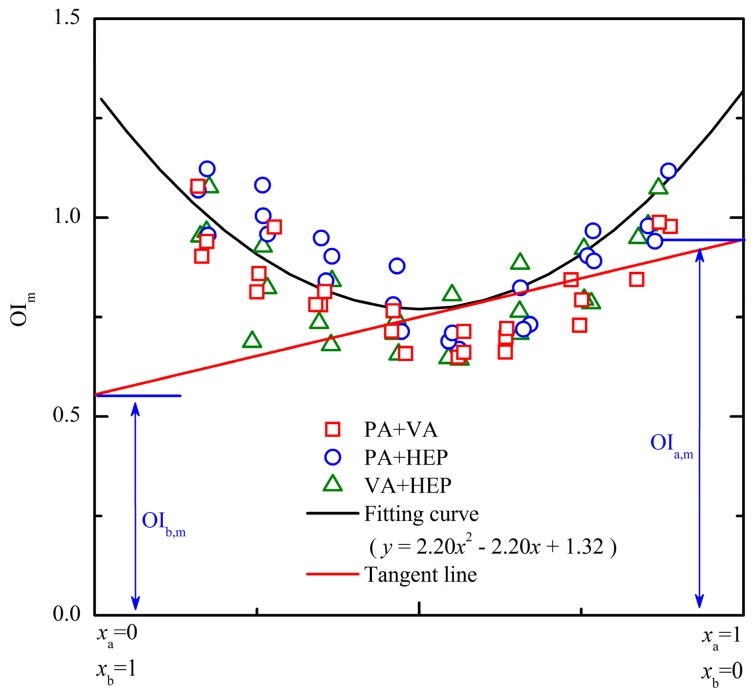
The extended PDE model for binary odor mixture of aldehydes. The red line was a tangent line on the fitting curve at point (0.6, 0.8), and its intercepts on the vertical axes were partial differential odor intensities (OI_a,m_ and OI_b,m_).

Based on the similarity of the three PDE models in Figure 4, their measurement results of every odor sample were gathered to form an extend PDE model. As depicted in Figure 6, all the odor samples in the extended PDE model apparently distributed like a “U” shape. After the quadratic polynomial regression analysis, the fitting equation of these odor samples was *y* = 2.20*x*^2^ − 2.20*x* + 1.32. The corresponding fitting curve was symmetric (symmetric axis was *x* = 0.5), and then the measurement of OI_i,m_ would become more convenient. For example, the fitting curve of binary mixture PA + VA (Figure 4a) would change if the horizontal axis was represented with *x*_VA_ instead of *x*_PA_. However, the fitting equation of the extended PDE model was fixed no matter the horizontal axis was represented with any component of a binary mixture. Because the OI_i,m_ represents the OI of each lnOAV unit substance actually contributed to the OI of a binary odor mixture. Then, the odor interaction between two aldehyde compounds could be directly observed from the extended PDE model. For instance, a binary odor sample with mixing ratio (e.g., lnOAV_PA_/lnOAV_VA_) of 0.3 was corresponded to the point (0.3, 0.86) on the fitting curve of the extended PDE model (Figure 6). Then, the tangent line at the point on the curve was *y* = −0.88*x* + 1.12. According to Equations (3) and (4), the measured results were OI_PA,m_ = 0.24 and OI_VA,m_ = 1.12. For another different binary odor mixture (e.g., (0.6, 0.79)), the results calculated with the same method were OI_PA,m_ = 0.97 and OI_VA,m_ = 0.53. Based on the above results, it was concluded that the influence of an odorant to the binary mixture's OI was in proportion to its proportion in the odor mixture.

#### 3.3.2. Predictive Performance of the Extended PDE Model

Besides the investigation of odor interaction, the extended PDE model also could be used for OI evaluation instead of human assessors. Three binary odor mixtures consisted of previously used aldehydes and four binary odor mixtures contained new aldehyde compounds were prepared to confirm the OI predictive performance of the extended PDE model. Four different odor samples of each odor mixture were prepared, and their odor intensities were individually rated by assessors in panel B (*i.e.*, OI_mea._, Table 2). Besides, chemical concentrations of the constituents in each binary odor sample were measured by a gas chromatography. Based on the measured chemical concentrations and the extended PDE model, the OI of each odor sample was also calculated according to Equation (6) (*i.e.*, OI_pre._).

As listed in Table 2, the predictive accuracy of the extended PDE model was quite good (e.g., average of OI_pre._/OI_mea._ was 1.03) among binary odor mixtures consisting of the previously used aldehydes. When the extended PDE model was applied to other odor mixtures, its predictive accuracy showed a small decrease. For example, the average of odor samples' predictive coefficients (*i.e.*, OI_pre._/OI_mea._) which indicates the predictive accuracy of the extended PDE model decreased from 1.03 to 0.93 (Table 2). However, the difference between OI_mea._ and OI_pre._ still belonged to random error for most of the odor sample. Although the specific PDE model of each odor mixture in this test was not individually explored, these odorants (e.g., BA, IBA, IVA and HEX) still fitted into the extended PDE model very well. It probably should be contributed to their same chemical group and similar molecular structure [24]. In a previous study, binary odor mixtures of benzene and substituted benzenes were also fitted into a same PDE model [10]. Thus, the laws of odor interaction were possibly very close to odorants with same chemical group and similar molecular structure.

## 4. Conclusions

In this research, the odor interactions between two aldehyde compounds were investigated by using the partial differential equation (PDE) model. The linear OI-lnOAV relation of individual odorant was tested for three different aldehyde compounds through olfactory evaluation by referencing to the odor intensity referencing scale (OIRS). Based on that, the binary odor mixtures of these three compounds were respectively prepared to explore their odor interaction in the form of 24 odor samples with different concentration levels and various mixing ratios. After the quantitative analysis and olfactory evaluation, the measurement results were processed to establish a PDE model for each binary odor mixture. Then, the effects of mixing ratio, concentration level and random error on the odor interaction of binary odor mixture were evaluated. Besides, an extended PDE model was also proposed for binary odor mixtures consisting of two aldehyde compounds. Through the comparison between the prediction value and olfactory measurement results, the extended PDE model was proved effective at OI prediction. In conclusion, the PDE model provided an interpretable way relating the chemical concentration of each component to their joint odor intensity. The laws of binary odor interaction were very similar among odorants with the same chemical group and similar odor type.

## Figures and Tables

**Table 1.** List of stimuli investigated for the PDE model.

**Order**	**Odorant (Abbreviation)**	**CAS#**	**Chemical Structure**	**Reported Odor Threshold/mg/m^3^** **(ppb)**	**Measured Odor Threshold**^III^**/mg/m^3^(ppb)**
1	Propionaldehyde (PA)	123-38-6	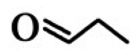	2.4E − 3 (1.0)^I^/65.0E − 3 (26.9)^II^	40.6E − 3 (16.8)
2	*n*-Butyraldehyde (BA)	123-72-8	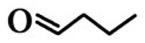	2.0E − 3 (0.67)^I^/26.7E − 3 (8.9)^II^	20.7E − 3 (6.90)
3	Isobutyraldehyde (IBA)	78-84-2	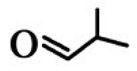	1.0E − 3 (0.35)^I^	48.4E − 3 (16.1)
4	*n*-Valeraldehyde (VA)	110-62-3	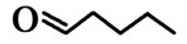	1.5E − 3 (0.41)^I^/21.6E − 3 (6.0)^II^	20.5E − 3 (5.71)
5	Isovaleraldehyde (IVA)	590-86-3	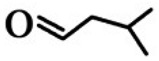	0.36E − 3 (0.10)^I^	21.7E − 3 (6.06)
6	*n*-Hexaldehyde (HEX)	66-25-1	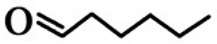	1.2E − 3 (0.28)^I^/57.5E − 3 (13.8)^II^	12.4E − 3 (2.98)
7	*n*-Heptaldehyde (HEP)	117-71-7	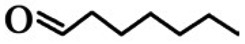	0.86E − 3 (0.18)^I^/22.7E − 3 (4.8)^II^	26.0E − 3 (5.47)

I Odor detection thresholds in reference [11];

II Odor detection thresholds in reference [12];

III Odor detection thresholds measured by panel A in this study.

**Table 2.** Odor intensity predictive performance of the extended PDE model.

**Mixture**	**Constitutions**		**OI_a,m_**	**OI_b,m_**	**OI_pre._**	**OI_mea._**

	**lnOAV_a_**	**lnOAV_b_**				

**A. Binary Odor Mixtures Consisted of Previously Used Aldehydes**						
a = PA; b = VA	2.18	0.56	1.32	–0.07	2.6	2.3
	3.09	2.87	0.81	0.73	4.6	4.3
	2.87	1.48	1.07	0.36	3.6	3.5

a = PA; b = HEP	0.57	1.95	0.00	1.21	2.4	2.3
	3.97	1.48	1.16	0.15	4.8	4.3
	2.87	1.48	1.07	0.36	3.6	3.8

a = VA; b = HEP	1.26	0.56	1.11	0.27	1.6	1.8
	2.87	3.27	0.70	0.84	4.7	4.8
	2.18	2.17	0.77	0.77	3.4	3.3

Average of OI_pre._/OI_mea._					1.03	

**B. Binary Odor Mixtures Contained Other Aldehydes**						

a = BA; b = HEX	2.16	0.78	1.16	0.14	2.6	2.7
	2.85	2.68	0.80	0.74	4.3	4.2
	2.16	4.29	0.35	1.07	5.4	4.8

a = BA; b = HEP	0.77	1.63	0.31	1.09	2.0	2.2
	2.16	1.98	0.81	0.72	3.2	2.9
	3.95	1.76	1.11	0.27	4.9	5.2

a = IVA; b = VA	0.90	1.95	0.29	1.10	2.4	2.8
	2.18	2.17	0.77	0.77	3.4	3.8
	2.87	2.17	0.91	0.61	3.9	5.0

a = IVA; b = IBA	1.88	0.60	1.19	0.06	2.3	2.7
	2.18	1.99	0.82	0.72	3.2	3.8
	1.48	3.37	0.26	1.11	4.1	4.7

Average of OI_pre._/OI_mea._					0.93	

## References

[1] Mizukoshi A., Kumagai K., Yamamoto N., Noguchi M., Yoshiuchi K., Kumano H., Yanagisawa Y. (2010). A novel methodology to evaluate health impacts caused by VOC exposures using real-time VOC and Holter monitors. Int. J. Environ. Res. Public Health.

[2] Osada K., Hanawa M., Tsunoda K., Izumi H. (2013). Evaluation of the masking of dimethyl sulfide odors by citronellal, limonene and citral through the use of trained odor sensor mice. Chem. Senses.

[3] Qamaruz-Zaman N., Milke M.W. (2012). VFA and ammonia from residential food waste as indicators of odor potential. Waste Manag..

[4] Kabir E., Kim K.H., Ahn J.W., Hong O.F., Chang Y.S. (2010). Offensive odorants released from stormwater catch basins (SCB) in an urban area. Chemosphere.

[5] Hansen M.J., Adamsen A.P., Pedersen P., Feilberg A. (2012). Prediction of odor from pig production based on chemical odorants. J. Environ. Qual..

[6] Kim K.H. (2011). The averaging effect of odorant mixing as determined by air dilution sensory tests: A case study on reduced sulfur compounds. Sensors.

[7] Miyazawa T., Gallagher M., Preti G., Wise P.M. (2008). Synergistic mixture interactions in detection of perithreshold odors by humans. Chem. Senses.

[8] Rodrigues A.E., Teixeira M.A., Rodriguez O. (2010). The Perception of Fragrance Mixtures: A Comparison of Odor Intensity Models. Aiche J..

[9] Cain W.S., Schiet F.T., Olsson M.J., de Wijk R.A. (1995). Comparison of models of odor interaction. Chem. Senses.

[10] Yan L., Liu J., Wang G., Wu C. (2014). An odor interaction model of binary odorant mixtures by a partial differential equation method. Sensors.

[11] Nagata Y. (2003). Measurement of Odor Threshold by Triangle Odor Bag Method. Odor Measurement Review.

[12] Zarzo M. (2012). Effect of functional group and carbon chain length on the odor detection threshold of alophatic compounds. Sensors.

[13] ASTM (2010). ASTM, E544–10. Standard Practices for Referencing Suprathreshold Odor Intensity.

[14] Walker J.C., Hall S.B., Walker D.B., Kendal-Reed M.S. (2003). Human odor detectability: New methodology used to determine threshold and variation. Chem. Senses.

[15] Ueno H., Higuchi M., Tatsuichi S., Iwasaki Y. (2003). A comparative study of Japanse and European olfactometry standards. Odor Measurement Review.

[16] Karnekull S.C., Jonsson F.U., Larsson M., Olofsson J.K. (2011). Affected by smell? Environmental chemical responsivity predicts odor perception. Chem. Senses.

[17] Aatamila M., Verkasalo P.K., Korhonen M.J., Viluksela M.K., Pasanen K., Tiittanen P., Nevalainen A. (2010). Odor annoyance near waste treatment centers: A population-based study in Finland. J. Air Waste Manag. Assoc..

[18] Brossard C., Rousseau F., Dumont J.P. (2007). Perceptual interactions between characteristic notes smelled above aqueous solutions of odorant mixtures. Chem. Senses.

[19] Wise P.M., Miyazawa T., Gallagher M., Preti G. (2009). Psychometric Functions for Ternary Odor Mixtures and Their Unmixed Components. Chem. Senses.

[20] Mori K., Takahashi Y.K., Igarashi K.M., Yamaguchi M. (2006). Maps of odorant melecular features in the mammalian olfactory bulb. Physiol. Rev..

[21] Thomas-Danguin T., le Berre E., Beno N., Ishii A., Chabanet C., Etievant P. (2008). Just noticeable differences in component concentrations modify the odor quality of a blending mixture. Chem. Senses.

[22] Lawson M.J., Craven B.A., Paterson E.G., Settles G.S. (2012). A computational study of odorant transport and deposition in the canine nasal cavity: Implications for olfaction. Chem. Senses.

[23] Scott J.W. (2006). Sniffing and spatiotemporal coding in olfaction. Chem. Senses.

[24] Lavine B.K., White C., Mirjankar N., Sundling C.M., Breneman C.M. (2012). Odor-structure relationship studies of tetralin and indan musks. Chem. Senses.

